# The relationship between socio-demographic factors, health status, treatment type, and employment outcome in patients with inflammatory bowel disease in Japan

**DOI:** 10.1186/s12889-017-4516-0

**Published:** 2017-07-04

**Authors:** J Mahlich, K Matsuoka, Y Nakamura, R Sruamsiri

**Affiliations:** 1 Health Economics, Janssen Pharmaceutical KK, 5-2, Nishi-kanda 3-chome Chiyoda-ku, Tokyo, 101-0065 Japan; 2 Düsseldorf Institute for Competition Economics (DICE), University of Düsseldorf, Düsseldorf, Germany; 3 Department of Gastroenterology and Hepatology, Tokyo Medical and Dental University, Tokyo, Japan; 4 Health Economics, Janssen Pharmaceutical KK, Tokyo, Japan; 5 Center of Pharmaceutical Outcomes Research, Naresuan University, Phitsanulok, Thailand

**Keywords:** Burden of disease, Inflammatory bowel disease, Japan, Unemployment

## Abstract

**Background:**

Inflammatory Bowel Disease (IBD) constitutes a huge burden for patients and studies show that IBD patients have difficulties remaining in employment. Because there is no data about the unemployment of IBD patients in Japan.

**Methods:**

We surveyed a representative sample of 1068 Japanese IBD patients regarding their employment status.

**Results:**

We found that the labor force participation rate is lower and unemployment higher for patients with IBD compared to the general population. Factors associated with unemployment in the IBD sample are older age, female gender, and the prevalence of depression.

**Discussion:**

IBD constitutes a high burden for patients in Japan regarding employment outcome.

## Background

In Japan, the Ministry of Health Labor and Welfare reports that there are 140,000 patients with Ulcerative Colitis (UC) and 40,000 with Crohn’s Disease (CD). However, patients with mild to moderate Inflammatory Bowel Disease (IBD) are probably not covered by these numbers and the actual numbers of IBD patients in Japan are estimated to be 20% to 40% higher [1]. According to a nationwide epidemiological survey in Japan in 1991, the annual incidence rate of CD was 0.51 per 100,000 population and for UC it was 18.1 per 100,000 population [2]. It was reported that the incidence of CD in Japan had quadrupled and the prevalence had increased by 4.7-fold from 1986 to 1998 [3]. Although the mortality of IBD is relatively small with a 10-year cumulative survival rate of 96% in patients with UC [4] and 96.9% in patients with CD in Japan [5], IBD constitutes a significant burden to patients. A significant negative correlation was demonstrated between a short-form (SF)-8 score and the degree of CD and UC symptoms in Japan [6]. Significantly impaired domains included physical functioning, role physical, vitality, social functioning, role emotional, and mental health.

Based on a survey of current workers with IBD in Japan, it is known that the symptoms of IBD significantly affect the patients’ ability to perform their work-related duties, thereby decreasing their work motivation and increasing the risk of depression [7]. The symptoms of IBD such as abdominal pain or bloody diarrhea have far reaching consequences on daily activities and have a negative impact on quality of life and work [8, 9].

It is widely known that IBD patients have a higher risk of unemployment. For example, studies in Canada showed that patients with IBD were likely to be unemployed [10]. A US based study also reported that patients with IBD had higher levels of unemployment, and that education plays a significant role in the employment outcomes [11]. In Europe, several studies reported that patients with IBD were more likely to be unemployed compared with the general population [10].

In Japan, however, there is no comprehensive evidence demonstrating the relationship between socio-demographic factors, health status, treatment type, and unemployment status in patients with IBD. The objective of this research was to examine the work status of Japanese IBD patients and to compare their employment situation with the general population. In addition, we aimed to identify the major factors that influence the employment status of patients with IBD in Japan.

Cultural attitudes towards employment in Japan differ from those in Western countries and therefore a Japan specific study is warranted. First, as recognized by many economists, the Japanese labor market is characterized by a high degree of employment security and corporate loyalty by both white-collar and blue-collar employees. Empirical research emphasizes the continued resilience of the so called “lifetime employment” system [12, 13]. A second difference to Western countries is the strong Japanese work ethic that leads Japanese workers to be less likely to call in sick and to view missed work more negatively than employees from other countries [14]. Indeed Japanese employees’ willingness to work at the expense of their own health is so extreme that there is a word in Japanese, karōshi, which translates to “occupational death” or “working oneself to death” [15]. Institutional differences in the social security systems are a third reason to have a fresh look at Japan. Compared to Western countries unemployment benefits are much lower and would incentivize employees to stay in employment as long as possible [16]. These cultural and institutional differences suggest that Japanese IBD patients might not be affected by unemployment as much as their Western peers.

## Methods

### Study population

We performed a nationwide online survey of 1068 Japanese IBD patients, filtered from a pool of 2,263,991 database members that expressed their willingness to participate in online surveys. The patients were asked for their basic clinical characteristics (diagnosis, age, gender), their socioeconomic status (marital status, income, educational level), their medical history (disease duration, surgical history, comorbidity) and treatment details (type of treatment, duration, type of hospital).

The primary study outcome was the employment status of patients with IBD. In the survey, patients were asked to classify their current work status. Responses to employment status included “work full time (7 or more hours a day, 5 days a week) outside the home”, “work part time outside the home”, “self-employed”, “full time house maker (house wife/house husband)”, “student”, “unemployed/pensioner”, and “other”. In the analysis, the responses were categorized as employed or unemployed. To achieve this, “employed status” included full-time workers, part-time workers, and self-employed workers. Education status was classified as either compulsory education or senior high school, vocational college or junior college, 4-year or 6-year university, or master’s degree.

The type of IBD was divided into “Crohn’s Disease” or “Ulcerative Colitis”. The time after the diagnosis of IBD was categorized and ranged from 0 to 2 years to longer than 15 years. We selected hyperlipidemia/dyslipidemia and hypertension as a physical comorbidity variable because they were observed in more than 10% of respondents (11.2% and 14.2%, respectively). Regarding mental comorbidities, depression was the only variable included in the survey questionnaires.

The current prescription of biologics and an experience of surgical treatment were asked as treatment variables. The interaction between the use of biologics and surgery experience was also examined. The outcome variable and explanatory variables are summarized in Table 1.

**Table 1:** Definition of variables used

Type of variable		Type of response
Outcome variable	Employment status	Unemployed
		Employed
Socio-demographic variables	Gender	Male
		Female
	Age	Years old
	Final education level	Compulsory education or senior high school
		Vocational college or junior college
		4-year or 6-year university
		Master’s degree
IBD variables	Type of IBD	Crohn’s Disease
		Ulcerative Colitis
	Time after the diagnosis of IBD	0–2 years
		3–8 years
		9–15 years
		Longer than 15 years
Comorbidity variables	Hyperlipidemia/dyslipidemia	No
		Yes
	Hypertension	No
		Yes
	Depression	No
		Yes
Treatment variables	Prescription of biologics	No
		Yes
	Experience of surgical treatment	No
		Yes
Interaction variables	Interaction between biologic and surgical treatment	Surgical treatment with biologics
		Surgical treatment without biologics

**Fig. 1:**
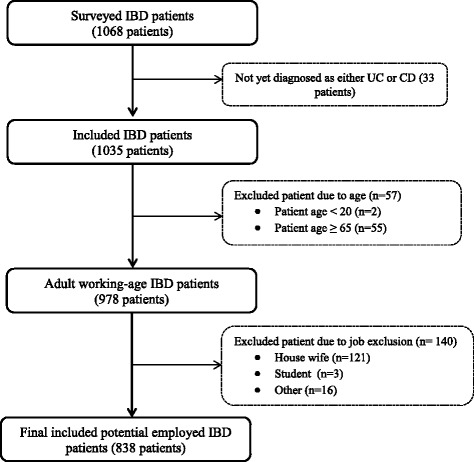
Study population

Figure 1 showed patient flow of this study. The sample ranged from 19 years to 83 years of age; 57 respondents aged either less than 20 years or more than 65 years old were excluded to analyze the adult working-age population. Moreover, we excluded 140 respondents who were full time housewives/housemen or students, to clearly define the employed status in the research. Therefore, the final sample included 838 patients. Because this was an online survey we cannot rule out the possibility of a selection bias towards people who are familiar with electronic devices who are typically more educated than the overall patient population.

### General Japanese population

To compare the employment status of IBD patients with that of the Japanese general population, we retrieved data regarding age distribution, gender, education status, and unemployment rate from several publicly available statistics published by Japanese government agencies [17–20]. Age distribution, gender and employment rate were obtained from reports in 2015, while education status was at the level of 2010 because of the lack of availability of more recent statistics.

### Statistical methods

Exploratory analyses were performed to compare the characteristics and outcome for the sampled patients with those for the Japanese general population. A bivariate logistic regression model was developed to estimate the odds ratios (ORs) for evaluating the risk factors associated with an employment status. There were no missing data in the analyzed population. Statistical significance was defined as *p* ≤ 0.1, which is a common threshold in real world studies [21]. All regression analyses were performed with STATA software version 12.0.

## Results

### IBD sample characteristics and comparison with the general Japanese population

The characteristics of the 838 included IBD patients are shown in Table 2. The sample included more patients with UC (75.7%) than with CD (24.3%). Of the total patients, 48% of patients were diagnosed as IBD more than 9 years ago, 24.1% had an experience of surgical treatment, and 17.7% were receiving biologic treatments. The majority of patients were male which is confirms a male predominance in epidemiological studies [22].

**Table 2:** Characteristics of the IBD patient sample versus the general population in Japan

Characteristics		Sample analyzed (n)	Sample analyzed (%)	Overall population (%)	Reference
Gender	Male	616	73.5%	48.6%	[18]
	Female	222	26.5%	51.4%	[18]
Age	20–29	53	6.3%	17.4%	[18]
	30–39	210	25.1%	21.8%	[18]
	40–49	331	39.5%	24.8%	[18]
	50–59	201	24.0%	20.9%	[18]
	60–64	43	5.1%	15.1%	[18]
Employment rate (Based on 735 employed patients)	20–29	46	75.4%	73.8%	[17]
	30–39	194	78.5%	80.8%	[17]
	40–49	299	78.7%	83.7%	[17]
	50–59	173	72.7%	81.1%	[17]
	60–64	23	44.2%	62.2%	[17]
Overall employment rate (age & gender adjusted)	20–64	838	75.1% (60.0%)	78.1%	[17, 18, 20]
Final education	Compulsory education or senior high school	221	26.4%	65.6%	[19]
	Vocational college or junior college	184	22.0%	14.7%	[19]
	4-year or 6-year university	369	44.0%	19.8%	[19]
	Master’s degree	64	7.6%		[19]
Unemployment	Unemployment rate	103	12.3%	3.2%	[20]
IBD type	Crohn’s Disease	204	24.3%	―	―
	Ulcerative Colitis	634	75.7%	―	―
Time after the diagnosis of IBD	0–2 years	137	16.3%	―	―
	3–8 years	299	35.7%	―	―
	9–15 years	186	22.2%	―	―
	Longer than 15 years	216	25.8%	―	―
Comorbidity	Hyperlipidemia/dyslipidemia	99	11.8%	―	―
	Hypertension	121	11.4%	―	―
	Depression	57	6.8%	―	―
Treatment	Prescription of biologics	148	17.7%	―	―
Stage	Experience of surgical treatment	202	24.1%	―	―

Also the age distribution of our sample resembles the overall Japanese IBD patient population with highest prevalence in the age cohort patients being in their 30’s and 40’s [23]. Although our survey was online based, this approach has apparently not resulted in an over-representation of younger patients.

Table 2 also shows a comparison of IBD patient sample characteristics with the general Japanese population. Compared to the general population, the age distribution of the sampled IBD patients was characterized by fewer younger people (20–29 years old) as well as fewer older people (60–64 years old) than the general population as of 2015. Regarding employment status, the proportion of university graduates was 2.6 times higher in the sampled IBD patients (51.6%) than in the general population (19.8%) as of 2010. The unemployment rate was approximately 4 times higher in the sampled IBD patients (12.3%) than in the general population (3.2%) as of 2015.

The employment ratio was consistently lower for IBD patients in all age cohorts. The gap was biggest for people over 50 years of age. Overall, the employment ratio in our sample was 75.1% as compared to 78.1% in the general population. To account for the different age and gender distribution, we also calculated the age adjusted rate for the UBD sample. After adjustment this rate is down to 60.0%. The big difference between the unadjusted and adjusted employment rate is mainly due to the large share of male patients in our sample. The adjustment accounts for the fact that males have a much higher employment rate than women in Japan.

### Determinants of employment status among IBD patients

Table 3 shows the results of the bivariate logistic regression analysis to determine the employment status. Coefficients were expressed as ORs. An OR greater than 1 indicated a higher likelihood of being employed and vice versa. Of the factors examined, higher education status (i.e. 4-year or 6-year university, or master’s degree), status of UC, and experience of surgical treatment were associated with an employed status. In contrast, older age, female, comorbidity of depression, and prescription of biologics were associated with an unemployed status. As there is a correlation between disease state and the use of biologics that might bias the results, we controlled for the disease state. Therefore, we used another variable (surgery) that is apparently related to the disease state and interacted this with the use of biologics. This method gives us the effect of biologic use given that a person is in a late stage of disease as indicated by previous surgery. The interaction term, “surgical treatment without biologics” was associated with a significantly lower employed status than that with “biologics”.

**Table 3:** Logistic regression of employment versus unemployment

Variable	OR of Employment	*P* value	[95% Confidence Interval]
Gender			
Male	(Reference)		
Female	0.62	0.07	[0.37–1.05]
Age			
One additional age	0.94	0.00	[0.91–0.96]
Final education level			
Compulsory or senior high school	(Reference)		
Vocational School	1.25	0.51	[0.65–2.38]
Junior college	1.11	0.80	[0.48–2.57]
4-year or 6-year university	2.21	0.00	[1.29–3.80]
Master’s degree	2.95	0.06	[0.97–8.91]
Type of IBD			
Crohn’s Disease	(Reference)		
Ulcerative Colitis	1.95	0.03	[1.06–3.59]
Time after the diagnosis of IBD			
0–2 years	(Reference)		
3–8 years	1.75	0.12	[0.86–3.56]
9–15 years	1.02	0.95	[0.50–2.09]
Longer than 15 years	1.10	0.79	[0.54–2.24]
Comorbidity			
Hyperlipidemia/dyslipidemia	1.31	0.52	[0.58–2.97]
Hypertension	0.74	0.38	[0.38–1.44]
Depression	0.29	0.00	[0.14–0.59]
Treatment			
Prescription of biologics	0.51	0.07	[0.25–1.06]
Experience of surgical treatment	2.44	0.08	[0.90–6.62]
Stage			
Interaction			
Experienced surgery			
Surgical treatment with biologics	(Reference)		
Surgical treatment without biologics	0.38	0.09	[0.12–1.17]
Constant term	59.44	0.00	[8.51–414.94]

OR, odds ratio

## Discussion

We found that a higher burden of unemployment was imposed on patients with IBD compared with the Japanese general population. This finding is consistent with other studies that report high unemployment rates among patients with various chronic conditions such as HIV [24, 25], cancer [25], mental health problems, and other illnesses [26, 27]. In this study, the unemployment rate was 12.3% for patients with IBD and 3.2% for the general population. The higher unemployment rate in patients with IBD is consistent with all burden on illness studies performed in the US [10, 11]. In contrast, a study from Denmark did not report any differences in unemployment rates [28]. Differences in health and social welfare systems and the economic situation might explain the discrepancy between this study and ours. For example, the unemployment rate in the Danish general population was already very high during the observation period (12.6%) compared with that in the US (5.0%) [29] or Japan (3.2%) [20]. Although we could not control for all differences in sample composition due to data availability, we do believe that the differences in employment rates would persist. This is because the sampled IBD patients are on average better educated then the overall Japanese population and education in turn is a major driver of the employment status. Our age and gender adjusted- employment rate for instance revealed a difference of 18% points between our sample of IBD patients and the overall population. Our results suggest that elderly IBD patients in particular find it difficult to remain in employment. This result is in line with results of a recent survey of Japanese patients where 35.5% of Japanese patients reported a job loss because of IBD [30]. Contrary to our initial hypothesis, the institutional and cultural context of Japan has not helped to cushion the adverse effects of the disease on employment outcomes. This finding is at odds with a recent Japanese survey of patients suffering from any kind of chronic diseases living in Miyagi prefecture. The authors of this study found an association between medical treatment for chronic disease and unemployment risk only in participants with a higher degree of psychological distress [31].

Several risk factors were also found to be associated with the employment status of IBD patients. In particular, older age, female gender, and depression were significant attributes of unemployed IBD patients. Depression is considered both as a possible cause as well as a symptom of IBD [32]. However, the relationship between unemployment and current treatment is more complex. According to the diagnosis and treatment guidelines for UC and CD, biologics are recommended for use in intractable cases of UC and severe cases of CD [33]. Surgery is considered for severe or extremely severe patients with UC and severe patients with CD [33]. After controlling for disease state we found that among those patients who had previous surgery, the use of biologics was associated with lower unemployment. However, we had no information regarding the date of surgery, which is a notable limitation of our study. Another limitation of this study was its cross-sectional design. Because treatment methods often change over time the interpretation of the coefficients of the medication term is surrounded by a high degree of uncertainty. Also, we could not define the clinical status or the severity of each IBD condition. However, a history of surgery correlates with disease severity measured as digestive damage especially in CD patients [34].

Future research on Japanese IBD patients should not only measure its effects on employment but also the productivity losses of those IBD patients who stay in employment but need to take sick leave. In Europe, for example, employed IBD patients have to take an annual sick leave of 3–6 weeks [35]. To better understand patient needs and develop effective support for Japanese IBD patients, further studies are required.

## Conclusion

Patients with IBD bear a large burden of unemployment in Japan that is almost four times that of the general population. Determinants of unemployment status in patients with IBD include older age, female gender, and comorbidity of depression. The relationship between unemployment and biologic treatment is more complex. Because biologics are used in late line treatments we analyzed the interaction of previous surgery and biologic treatment. This interaction effect indicated that treatment with biologics after surgery helped patients to remain in employment. This is an exploratory study that warrants validation in a more controlled setting. We believe that this research will help clinicians and policy makers develop an effective intervention to improve the societal participation of patients with IBD in Japan.
